# Assessing oral health knowledge among public school children in Saudi Arabian subpopulation

**DOI:** 10.1186/s41043-024-00617-0

**Published:** 2024-08-19

**Authors:** Sultan Abdulrahman Almalki, Abdul Habeeb Adil, Mohammed Mustafa, Mohmed Isaqali Karobari

**Affiliations:** 1 Department of Preventive Dental Sciences, College of Dentistry, Prince Sattam Bin AbdulAziz University, Al‑Kharj, 11942 Saudi Arabia; 2 Department of Dental Research, Saveetha Medical College and Hospital, Saveetha Institute of Medical and Technical Sciences, Saveetha University, Chennai, Tamil Nadu 602105 India; 3 Department of Conservative Dental Sciences, College of Dentistry, Prince Sattam Bin Abdulaziz University, Al-Kharj, 11942 Saudi Arabia; 4 Department of Restorative Dentistry and Endodontics, Faculty of Dentistry, University of Puthisastra, Phnom Penh, 12211 Cambodia

**Keywords:** Awareness, Knowledge, Dental, Oral health, Oral hygiene habits, School-going children, Oral hygiene educational, Saudi Arabia

## Abstract

**Introduction:**

Oral hygiene is paramount for maintaining optimal oral and physical health, especially among children who are particularly susceptible to dental caries and issues due to dietary habits and inadequate hygiene practices. This study aimed to evaluate the awareness and knowledge of oral health among public school children, analyse their oral hygiene habits, educate parents on the importance of oral health for their children, and implement an oral hygiene educational program in Al-Kharj City, Saudi Arabia.

**Methods:**

Four public elementary schools were selected for the study, with a sample size of 200 school-going children participating. A structured questionnaire was used to assess awareness and knowledge of oral health among the participants. SPSS software version 26 was used for data analysis. Multiple regression analysis and correlation tests was employed to explore the relationships between the different variables measured in the study.

**Results:**

The findings revealed a significant lack of awareness regarding oral health among school children. Oral hygiene habits were found to be inadequate in many participants. Overall, the findings highlight the need for targeted interventions aimed at promoting regular dental visits, addressing dental fear, and fostering positive oral hygiene practices among school-going children to improve oral health outcomes in the studied population.

**Conclusion:**

The study highlighted a critical gap in oral health awareness among school children, underscoring the need for targeted interventions. While the oral hygiene educational program has been implemented and future data will shed light on its effectiveness, preliminary observations suggest that such programs could potentially improve oral health outcomes and overall engagement among students.

## Introduction

The oral cavity is one of the most significant structures of the human body, which has a direct impact on the oral and physical health of an individual [1, 2]. The primary function of the oral cavity is to initiate the digestion process and to break down food into carbohydrates through the enzymatic reaction caused by the enzymes in the saliva [3] however, it has a secondary function too, which is to defend against the bacteria that might enter the body with the food eaten by that individual [4]. For the fulfilment of the breakdown of food and to defend against bacteria, it is essential to have a complete and functional oral cavity [5]. However, several diseases and medical conditions that are there can affect the functioning of the oral cavity partially and can lead to various other medical conditions. These medical conditions affecting the oral cavity and its functioning are usually caused by the improper eating habits of children of school-going age [6]. These children usually aged between 3 to 15 years are mostly affected by these diseases namely dental caries, tooth abscesses, teeth loss, etc., a large number of children are affected usually by these diseases of the oral cavity [7, 8].

Dental caries is one of the most common of all oral cavity-related dental issues [9]. Moreover, the study also provides evidence that these dental issues are more prevalent in children of school-going age, especially in the populations, which are socioeconomically deprived [10]. The evidence suggests that these issues are usually caused by improper diet and the lack of awareness in these populations [11].[21] In the world, around 60 to 90% of school-going children have dental cavities and nearly almost every adult has dental cavities according to a survey conducted by the World Health Organization. Oral diseases pose a major health burden for many countries and affect people throughout their lifetime, causing pain, discomfort, disfigurement, and even death. Most oral health conditions are largely preventable and can be treated in their early stages [12].

Various research has provided evidence that the maintenance of oral health could be helpful for children and adults to reduce the risk of various diseases as the oral cavity not only provides the ability to eat but also is the first line of defence against bacteria entering the body through food [13, 14]. Studies have shown that the oral cavity is strongly linked with diverse aspects of life and various oral medical conditions are directly related to the lifestyle of the individual and the eating habits [15]. The contaminated nature of the oral cavity will increase the risk of oral diseases and infections and could lead to various other medical conditions especially related to the gastrointestinal tract and various other life-threatening medical conditions [16, 17].

Various studies have supported the idea that by increasing awareness through educational programs and by providing free or cost-effective initial treatments the medical issues associated with the oral cavity and its prevalence can be decreased significantly [18–20]. These studies focus on the idea that providing health-related education to the parents of the children about the eating habits of their children can be improved which could improve the situation significantly. Oral health diseases have emerged as a major challenge for healthcare professionals in recent years [21]. In Saudi Arabia, it has been analysed and evaluated that the incidence rate of oral diseases is increasing continuously [22]. In particular, Saudi children are extremely prone to the risk of oral diseases because of improper oral hygiene habits [23]. The involvement of children in dental or oral diseases is affecting their educational careers and social lifestyles [24]. The quality of life is also affected by these oral health issues [25]. The use of positive and constructive oral hygiene habits will help improve the oral health of the public school-going children in the region of Al-Kharj, Saudi Arabia [26].

Several studies, both in developing and developed countries, have underscored the significance of oral health, particularly among school-going children. For example, a study investigating the impacts of impaired oral health on school performance and attendance revealed alarming trends in the global prevalence of oral diseases among children [27]. The findings of this study indicated that children with poor oral health were absent from school three times more frequently than their healthy counterparts [28]. This highlights the detrimental effects of impaired oral health on children's academic outcomes and attendance rates.

In India, studies focused on assessing the knowledge and attitudes of school children toward oral hygiene shed light on the importance of oral health maintenance for achieving educational objectives effectively [18, 19]. The study revealed that many schoolchildren lacked awareness and understanding of oral hygiene practices. Consequently, the studies emphasized the need for children to acquire appropriate knowledge and information to address oral hygiene issues effectively and promote better oral health outcomes. These findings underscore the importance of education and awareness initiatives targeting school children to instil positive oral hygiene habits early in life [18, 19]. By addressing the knowledge gaps and promoting positive oral hygiene behaviours, this study aimed to assess the awareness and knowledge of oral health among public school children, examine their oral hygiene habits, educate parents about the importance of oral health for their children, and implement an oral hygiene educational program in Al-Kharj City, Saudi Arabia.

## Methodology

### Study design

This study employed a cross-sectional survey design to assess the oral health awareness, perceptions, and behaviours of school-going children in Al-Kharj City, Saudi Arabia. A quantitative approach was utilised to gather data through structured questionnaires administered to the participants.

### Study setting

The study targeted public elementary schools in Al-Kharj City, Saudi Arabia. Specifically, four schools were selected as study sites: Ali bin Abi Talib School, Musab bin Omair School, Sa'ad Ibn Mu'adh School, and Riyadh School. The selection schools in Al-Kharj City for this study was based on a combination of factors including geographical representation, school size, and willingness to participate. These schools were chosen to provide a diverse and representative sample of the school-going population in the area, ensuring that the findings would be generalizable to a broader context. Additionally, logistical considerations such as accessibility and existing relationships with school administrations facilitated the selection process.

### Participants

The inclusion criteria for participants were children aged between 6 to 12 years who were enrolled as students in the selected schools, irrespective of their grade levels. Exclusion criteria were applied to students undergoing oral health treatment at the time of the study to avoid potential confounding effects on the study outcomes.

### Sampling

A convenience sampling method was utilised to select participants from each school. The sample size was calculated using the Raosoft sample size calculator with 95% CI, 5% margin of error and 50% response distribution, approximately 200 students were included in the study, with approximately 50 students aged 6 to 8 years and approximately 150 students aged 9 to 12 years.

### Data collection

Data collection for this study was conducted using a structured questionnaire (Table 1) before the implementation of oral hygiene education programme. The questionnaire was designed to gather information on various aspects of oral health awareness, perceptions, and behaviours among school-going children in Al-Kharj City, Saudi Arabia. The questionnaire was administered to the participants in the selected schools during the study period. To ensure the validity and reliability of the questionnaire, several methodologies used in previous studies were incorporated into the data collection process.

**Table 1:** Structured questionnaire

Questionnaire		
Q1. How many times do you clean your teeth daily?		
1. Once	2. Twice	3. Thrice
Q2. Who helps you when you brush your teeth?		
1. Mother	2. Father	
Q3. Did you clean your teeth by using toothpaste and a toothbrush?		
1. Yes	2. No	
Q4. What do you think cleaning your teeth by using a toothbrush and toothpaste will help you in preventing oral diseases?		
1. Yes	2. No	
Q5. Do you use dental floss after brushing your teeth?		
1. Yes	2. No	
Q6. Do you visit the dentist, if yes then how frequently?		
1. Once in Six months	2. Once a Year	
Q7. Have you ever felt fear while visiting the dentist?		
1. Yes	2. No	
Q8. What was the reason behind fear during the first visit to the dentist’s clinic?		
1. You fear that it is going to be painful	2. Are you afraid of the dentist?	
Q9. Do you consider your teeth like any other part of your body?		
1. Yes	2. No	

### Questionnaire development

The questionnaire was developed based on established scales and validated instruments used in previous studies assessing oral health awareness and behaviours among children. Items were adapted from validated questionnaires that have demonstrated reliability and validity in similar populations. Additionally, input from experts in the field of paediatric dentistry and public health was sought to ensure the questionnaire's relevance and comprehensiveness.

### Pilot testing

Before the main data collection, a pilot test of the questionnaire was conducted with a small sample of school children from a different school in Al-Kharj City. The purpose of the pilot test was to assess the clarity, comprehensibility, and appropriateness of the questionnaire items. Feedback from the pilot test participants was used to refine and finalise the questionnaire before its administration to the main study sample.

### Administration of questionnaire

The questionnaire was administered to the participants in a classroom setting by trained research assistants or school staff members. Before administering the questionnaire, the purpose of the study and the importance of providing accurate and honest responses were explained to the participants to encourage their cooperation and minimize response bias. Participants were assured of the confidentiality and anonymity of their responses to promote openness and honesty.

### Data quality control

To ensure the quality and completeness of the data collected, measures were implemented to monitor the data collection process. Research assistants or school staff members overseeing the administration of the questionnaire provided guidance and clarification to participants as needed to ensure that all items were understood and completed accurately. Additionally, periodic checks were conducted to verify the completeness and accuracy of the collected data.

### Data management

Completed questionnaires were collected and securely stored for subsequent data entry and analysis. To minimize errors during data entry, double-entry verification procedures were employed, where data entered into the database were independently verified by a second researcher to ensure accuracy and consistency.

### Ethical considerations

Ethical approval for the study was obtained from the ethical committee of the College of Dentistry, Prince Sattam Bin AbdulAziz University, Al-Kharj, under approval number COD/EC/10/2021–22. Written consent was obtained from the heads of the selected schools and the parents or legal guardians of all participating children. Measures were implemented to protect participant confidentiality and ensure non-discrimination throughout the study.

### Data analysis

SPSS software version 26 was used for data analysis. Descriptive statistics, including frequencies, percentages, means, and standard deviations, were used to summarize the demographic characteristics and responses to the questionnaire items. Multiple regression analysis was employed to explore the relationships between oral hygiene habits, dietary practices, and dental care utilization, and their impact on children's oral health outcomes. Correlation tests were conducted to assess the strength and direction of relationships between different variables measured in the study.

## Results

Table 2 shows descriptive statistics of various aspects of oral hygiene behaviours and attitudes as reported by the participants. It was found that the majority of children clean their teeth approximately once a day, with both parents commonly assisting them during brushing sessions. Additionally, most children reported using toothpaste and a toothbrush for oral hygiene maintenance, while a smaller proportion mentioned the use of miswak. Notably, all students recognized brushing with toothpaste and toothbrush as an effective preventive measure against oral diseases. However, the utilization of dental floss after brushing was reported by only a minority of students. Furthermore, the average frequency of dental visits among the participants was found to be suboptimal, indicating a potential gap in dental care utilization. Many children expressed fear or anxiety about visiting the dentist, underscoring the importance of addressing misconceptions and promoting a positive attitude toward dental care. Despite these challenges, the vast majority of children acknowledged the importance of their teeth as vital body parts. While most children perceived themselves as healthy, reports of gum or tooth pain suggest the presence of underlying oral health issues requiring attention.

**Table 2:** Descriptive statistics

	N	Minimum	Maximum	Mean	Std. deviation
How many times do you clean your teeth daily?	200	.00	3.00	2.0800	.80426
Who helps you when you brush your teeth?	200	1.00	2.00	1.5000	.50125
Did you clean your teeth by using toothpaste and a toothbrush?	200	0.00	2.00	2.000	.50125
What do you think cleaning your teeth by using toothbrushes and toothpaste will help you in preventing oral diseases?	200	1.00	2.00	1.0000	.00500
Do you use dental floss after brushing your teeth?	200	1.00	2.00	1.0500	.21849
Do you visit the dentist, if yes then how frequently?	200	1.00	2.00	1.0300	.50035
Have you ever felt fear while visiting the dentist?	200	1.00	2.00	1.0000	.05000
What was the reason behind fear during the first visit to the dentist’s clinic?	200	1.00	2.00	1.0000	.5000
Do you consider your teeth like any other part of your body?					
	200	.00	1.00	.0000	.0057
Oral Health	200	.00	1.00	1. 00	.0005
Valid N (list-wise)	200				

Table 3a depicts the multiple regression analysis, which shows a strong relationship between oral health and children's awareness of oral health. The regression model (Model 1) showed a high coefficient of determination (R Square = 0.815), indicating that approximately 81.5% of the variance in oral health outcomes could be explained by the predictor variables included in the model. The model's overall fit was strong (R = 0.903), suggesting a robust relationship between the predictor variables and oral health outcomes. The predictors included in the model were frequency of dental visits, utilization of toothpaste and toothbrushes for cleaning teeth, assistance received during tooth brushing, use of dental floss after brushing, and frequency of teeth cleaning per day. Notably, the frequency of dental visits emerged as a significant predictor, highlighting the importance of regular dental check-ups in maintaining oral health. Overall, these findings underscore the critical role of oral health awareness and hygiene practices in promoting better oral health outcomes among school-going children.

**Table 3:** Multiple regression analysis

Model	R	R square	Adjusted R square	Std. error of the estimate
a. Model summary				
1	0.903^a^	0.815	− 0.011	0.28844

Model	Sum of squares	df	Mean square	F	Sig
b. ANOVA ^b^					
1 Regression	0.240	5	0.048	0.577	0. 72^a^
Residual	16.140	194	0.083		
Total	16.380	199			

Model	Unstandardized coefficients		Standardized coefficients	t	Sig
	B	Std. error	Beta		
c. Coefficients^a^					
1. (Constant)	0.811	0.165		4.912	0.000
How many times do you clean your teeth daily?	− 0.002	0.026	− 0.006	− 4.089	0. 000
Who helps you when you brush your teeth?	− 0.021	0.041	− 0.036	− 4.505	0. 000
Did you clean your teeth by using toothpaste and a toothbrush?	0.042	0.041	0.073	14.015	0. 000
Do you use dental floss after brushing your teeth?	− 0.008	0.094	− 0.006	− 4.083	0. 000
Do you visit the dentist monthly, if yes then how frequently?	0.053	0.041	0.092	4.280	0. 000

The analysis of variance (ANOVA) results for the regression model are presented in Table 3b. The model's ability to explain the variance in the dependent variable (oral health) was assessed through the examination of the regression sum of squares, residual sum of squares, and total sum of squares. The regression sum of squares (0.240) represents the amount of variance in the dependent variable explained by the regression model. The residual sum of squares (16.140) reflects the unexplained variance in the dependent variable. The total sum of squares (16.380) represents the overall variability in the dependent variable. The F-statistic value of 0.577 tests the overall significance of the regression model. With a significance level of 0.72a, the p-value associated with the F-statistic is greater than the defined level of significance, indicating that the regression model is not statistically significant. This suggests that the predictors included in the model may not collectively contribute to the variance in the dependent variable. Furthermore, the value of 0.72 provides evidence supporting the hypothesis, suggesting a positive relationship between the predictor variables (frequency of dental visits, utilization of toothpaste and toothbrush, assistance during tooth brushing, use of dental floss, and frequency of teeth cleaning) and oral health outcomes. Therefore, the null hypothesis is rejected in favour of the alternative hypothesis.

The coefficients for the predictors in the regression model are presented in Table 3c. These coefficients provide information about the magnitude and direction of the relationship between each predictor variable and the dependent variable (oral health). The unstandardized coefficients (B) represent the change in the dependent variable (oral health) associated with a one-unit change in each predictor variable. The standardized coefficients provide a measure of the relative importance of each predictor variable, allowing for comparisons between predictors with different scales. The t-values assess the significance of each coefficient, with higher absolute t-values indicating greater significance. The p-values associated with each coefficient indicate whether the relationship between the predictor variable and the dependent variable is statistically significant. Overall, the coefficients indicate that frequency of teeth cleaning, assistance during tooth brushing, utilization of toothpaste and toothbrush, use of dental floss, and frequency of dental visits are all significant predictors of oral health outcomes among the study participants. These findings highlight the importance of oral hygiene practices and regular dental care in maintaining optimal oral health among school-going children.

The correlation analysis presented in Table 4 examines the relationships between various oral health-related variables among the study participants. The correlation coefficients and their significance are reported for each pair of variables. Correlation coefficients range from -1 to 1, with positive values indicating a positive relationship, negative values indicating a negative relationship, and values close to zero indicating no significant relationship. The results indicate there is a weak positive correlation between the frequency of teeth cleaning and oral health (r = 0.012, *p* = 0.864). No significant correlations were found between other oral health-related variables and oral health outcomes, such as the assistance received during tooth brushing, utilization of toothpaste and toothbrush, use of dental floss, and frequency of dental visits.

**Table 4:** Correlation

		How many times do you clean your teeth daily?	Who helps you when you brush your teeth?	Did you clean your teeth by using toothpaste and a toothbrush?	What do you think cleaning your teeth by using a toothbrush and toothpaste will help you in preventing oral diseases?	Do you use dental floss after brushing your teeth?	Do you visit the dentist, if yes then how frequently?	Oral Health
How many times do you clean your teeth daily?	Pearson Correlation	1	0.012	0.075	.^a^	− 0.023	− 0.118	− 0.012
	Sig. (2- tailed)		0.861	0.293		0.748	0.095	0.864
	N	200	200	200	200	200	200	200
Who helps you when you brush your teeth?	Pearson Correlation	.012	1	− 0.040	.^a^	− 0.092	0.040	− 0.035
	Sig. (2- tailed)	0.861		0.574		0.196	0.573	0.623
	N	200	200	200	200	200	200	200
Did you clean your teeth by using toothpaste and a toothbrush?	Pearson Correlation	0.075	− 0.040	1	.^a^	0.000	− 0..040	0.070
	Sig. (2- tailed)	0.293	0.574			1.000	0.573	0.325
	N	200	200	200	200	200	200	200
Do you use dental floss after brushing your teeth?	Pearson Correlation	− 0.023	− 0.092	0.000	.^a^	1	− 0.060	− 0.008
	Sig. (2- tailed)	0.748	0.196	1.000			0.401	0.910
	N	200	200	200	200	200	200	200
Do you visit the dentist, if yes then how frequently?	Pearson Correlation	− 0.118	0.040	− 0.040	.^a^	− 0.060	1	0.089
	Sig. (2- tailed)	0.095	0.573	0.573		0.401		0.211
	N	200	200	200	200	200	200	200
Oral Health	Pearson Correlation	− 0.012	− 0.035	.070	.^a^	− 0.008	0.089	1
	Sig. (2- tailed)	0.864	0.623	0.325		0.910	0.211	
	N	200	200	200	200	200	200	200

## Discussion

A significant number of studies have been conducted in developing and developed countries, which have focused on the importance of oral health [29–32]. A study was conducted by Jackson et al. (2011) which described the impacts of impaired oral health on the performance and attendance of school-going children. The study has described that the incidence rate of oral diseases is continuously increasing among children globally. The findings of the study mentioned that children having poor oral health, missed their school three (03) times as compared to healthy children. Therefore, it can be concluded that impaired oral health will affect the performance and attendance of school-going children [28].

In the present study, the majority of children reported practising daily teeth cleaning, often with assistance from their parents, and predominantly using toothpaste and toothbrushes for oral hygiene maintenance. Interestingly, while the use of miswak was less common, all students recognized the importance of toothpaste and toothbrushes in preventing oral diseases, indicating a widespread understanding of basic oral hygiene practices. However, the study also revealed several areas of concern. Despite the recognized importance of dental hygiene, the utilization of dental floss, a key component of comprehensive oral care, was reported by only a minority of students. Moreover, the average frequency of dental visits was found to be suboptimal, suggesting a gap in accessing routine dental care among the study participants. The presence of fear or anxiety about visiting the dentist among many children underscores the need to address misconceptions and promote positive attitudes toward dental care from an early age.

Additionally, while the participants generally acknowledged the importance of their teeth as vital body parts, reports of gum or tooth pain suggest the existence of underlying oral health issues requiring attention. This discrepancy between perceived health and reported symptoms highlights the importance of comprehensive oral health assessments and regular check-ups to detect and address potential problems early on. Similar patterns of oral hygiene behaviours and attitudes have been observed among school-going children in various cultural and geographical contexts [33–35]. Studies conducted in different countries have consistently reported inadequate dental hygiene practices, low utilization of preventive dental services, and barriers such as fear or anxiety about dental visits among children [36–39]. These findings underscore the global nature of oral health challenges among children and the importance of implementing targeted interventions to address these issues effectively.

The overall fit of the regression model (R = 0.903) indicates a robust relationship between the predictor variables and oral health outcomes. This suggests that the included predictors, such as frequency of dental visits, utilization of toothpaste and toothbrushes, assistance received during tooth brushing, use of dental floss, and frequency of teeth cleaning per day, collectively contribute to better oral health outcomes among school-going children. Of particular note is the significant predictive power of the frequency of dental visits, emphasizing the importance of regular dental check-ups in maintaining oral health. This finding aligns with previous research [14, 19] highlighting the crucial role of preventive dental care in reducing the risk of oral diseases and promoting oral health among children. The fact that parents often assist their children during tooth brushing sessions reflects the importance placed on oral hygiene within the family context. Additionally, the utilization of traditional oral hygiene tools like miswak among some children suggests a diverse range of oral hygiene practices within the studied population. However, it's important to note that while the present study and the study by Batwala et al. (2007) both highlight the prevalence of oral health issues among school-going children, the specific challenges and barriers to oral health may vary across different contexts and populations. Factors such as socioeconomic status, access to dental care, cultural practices, and educational initiatives can significantly influence oral health outcomes among children.

The studies by Kuppuswamy et al. (2014) and Shivakumar et al. (2017) provide valuable insights into the knowledge, attitudes, and oral health status of school children in India, highlighting important issues that need to be addressed to improve oral health outcomes in this population. Kuppuswamy et al. (2014) emphasize the importance of oral health maintenance for children to achieve their objectives effectively. However, their study reveals a lack of awareness and knowledge among schoolchildren regarding oral hygiene practices. This underscores the critical need for educational interventions to equip children with the necessary information and skills to maintain good oral hygiene and prevent dental problems effectively [19]. Similarly, the study by Shivakumar et al. (2017) paints a concerning picture of oral health among school children in India, with findings indicating the increased prevalence of caries, poor oral hygiene, and a high need for dental treatment. The study suggests that these children may be neglected in terms of dental care, with lower treatment priority compared to other populations [40]. In the present study, similar trends were observed, with the majority of participants demonstrating limited awareness and knowledge about oral health habits and dental treatment needs. This suggests that the issues identified by Kuppuswamy et al. (2014) and Shivakumar et al. (2017) are not unique to their studies but are reflective of broader challenges within the population.

The regression sum of squares (0.240) indicates the amount of variance in the dependent variable that is explained by the regression model. This represents the portion of the variability in oral health outcomes that can be attributed to the predictor variables included in the model. A higher regression sum of squares suggests a stronger relationship between the predictor variables and oral health outcomes. Comparing these results with findings from other studies, Batwala et al. (2007) also assessed the variance in oral health outcomes among school-going children and highlighted the importance of addressing the unexplained variance through targeted interventions [14]. Similarly, Togoo et al. (2011) emphasized the significance of understanding the factors contributing to variability in oral health outcomes among children to inform effective preventive strategies [41].

The study by Togoo et al. (2011) conducted in the Abha region of Saudi Arabia emphasizes the significant impact of oral health status on individuals, particularly children, in achieving their personal and professional goals. The findings underscore the crucial role of oral health in maintaining overall well-being, as it influences metabolic and immune system functions and contributes to the developmental processes of children. The study highlights the importance of oral hygiene strategies in ensuring proper performance and overall health outcomes [41]. In contrast, the present study revealed a concerning lack of basic information and practice about oral health among the majority of participants. This discrepancy between the recognized importance of oral health in achieving personal and professional goals, as emphasized by Togoo et al. (2011), and the observed lack of awareness and practice in our study population underscores the need for targeted interventions to address gaps in oral health knowledge and behaviour.

The F-statistic value of 0.577 tests the overall significance of the regression model. With a significance level of 0.72a, the associated p-value is greater than the defined level of significance, indicating that the regression model as a whole is not statistically significant. This suggests that the included predictors may not collectively contribute to the variance in oral health outcomes. However, the individual coefficients for the predictor variables suggest a positive relationship between the frequency of dental visits, utilization of toothpaste and toothbrush, assistance during tooth brushing, use of dental floss, frequency of teeth cleaning, and oral health outcomes. The coefficient value of 0.72 provides evidence supporting this hypothesis, indicating that these factors may have a positive impact on oral health outcomes. Factors such as sample size, study design, and measurement methods can influence the results and should be considered when interpreting findings. Upon examination of the coefficients, it is evident that several predictor variables significantly influence oral health outcomes among the study participants. Specifically, the frequency of teeth cleaning, assistance during tooth brushing, utilization of toothpaste and toothbrush, use of dental floss, and frequency of dental visits emerged as significant predictors of oral health outcomes. The correlation coefficients suggest that there is a weak positive correlation between the frequency of teeth cleaning and oral health outcomes (r = 0.012, *p* = 0.864). This finding suggests that individuals who clean their teeth more frequently may have slightly better oral health outcomes. However, the correlation coefficient is close to zero, indicating a very weak association between these variables. Interestingly, no significant correlations were found between other oral health-related variables and oral health outcomes, including assistance received during tooth brushing, utilization of toothpaste and toothbrush, use of dental floss, and frequency of dental visits. These results suggest that these factors may not have a significant direct impact on oral health outcomes among the study participants.

The findings of this study suggest that despite the recognized significance of oral health, there may be barriers or challenges preventing individuals, particularly children, from effectively implementing oral hygiene strategies. Factors such as inadequate access to oral health education, limited resources, and cultural beliefs may contribute to this discrepancy. To bridge this gap, it is imperative to implement comprehensive oral health promotion programs that target children and their families, as well as the broader community. These programs should focus on raising awareness about the importance of oral health, providing practical information on oral hygiene practices, and promoting regular dental check-ups. Collaborative efforts involving healthcare professionals, educators, policymakers, and community leaders are essential to ensure the success of such initiatives. By addressing the underlying factors contributing to poor oral health knowledge and practice, we can empower individuals to take control of their oral health and improve overall well-being. This aligns with the goals outlined by Togoo et al. (2011) that underscores the importance of prioritizing oral health promotion efforts to support individuals in achieving their personal and professional aspirations.

The studies by Al-Jobair et al. (2013) and Mustafa et al. (2018) emphasize the oral health status and awareness levels among specific populations in Saudi Arabia, highlighting important considerations for promoting oral health and addressing gaps in knowledge and practice. Al-Jobair et al. (2013) focused on orphan children in Saudi Arabia and demonstrated the positive impact of delivering appropriate knowledge on oral health. Through educational interventions, the study effectively improved the attitudes and behaviours of the children, leading to the adoption of oral hygiene strategies and ultimately resulting in improved oral health status [26]. This underscores the importance of targeted oral health education programs, particularly for vulnerable populations such as orphan children, to empower them with the necessary knowledge and skills to maintain good oral health. In contrast, the study by Mustafa et al. (2018) examined the awareness levels regarding oral health and dental treatment needs among individuals with hearing and speech impairments in Saudi Arabia. The findings revealed a concerning lack of awareness and basic knowledge about oral health among this population [42]. This highlights the importance of tailored approaches to oral health education that address the unique needs and challenges faced by individuals with disabilities. Strategies such as using visual aids, sign language, and accessible materials can help improve understanding and promote oral hygiene practices among individuals with hearing and speech impairments.

The study conducted by Farsi et al. (2013) highlights the high risk of dental caries among children in Saudi Arabia and the effectiveness of oral hygiene tactics in addressing oral health problems. Through the implementation of oral hygiene strategies, the selected children were able to recover from a diverse range of oral and dental issues, highlighting the importance of preventive measures in managing oral health problems effectively [43]. In the present study, similar trends were observed, with the majority of students recognizing brushing as a preventive method against oral diseases. However, the utilization of dental floss was reported by only a small number of students, indicating a potential gap in oral hygiene practices among the participants. Furthermore, the average frequency of dental visits among the students was found to be suboptimal, with fear and lack of awareness acting as barriers to seeking dental care.

These findings underscore the importance of promoting oral hygiene practices and increasing awareness about the significance of regular dental check-ups among school children in Saudi Arabia. By addressing misconceptions and fears surrounding dental visits and emphasizing the importance of preventive measures such as brushing and flossing, we can work towards improving oral health outcomes and reducing the prevalence of dental caries among children. Collaborative efforts involving parents, educators, healthcare professionals, and policymakers are essential to implement effective oral health promotion programs that target school children and address the barriers to accessing dental care. By empowering children with the necessary knowledge and skills to maintain good oral hygiene and seek timely dental treatment, we can contribute to the overall well-being and oral health of the younger generation in Saudi Arabia.

The results of this study suggest that there is a concerning lack of basic knowledge about oral health among the students, which may contribute to increased school absenteeism. The findings highlight a potential gap in understanding among both students and their parents regarding the importance of oral health in improving overall physical health. Despite parents regularly assisting children with brushing, the low utilization of dental floss among children suggests a broader lack of awareness about oral hygiene practices, including the significance of dental floss in preventing oral diseases. The observed lack of basic knowledge about oral health among students and their parents underscores the need for comprehensive oral health education initiatives targeting both groups. By providing students and parents with the necessary information and skills to understand the importance of oral health and adopt appropriate oral hygiene practices, we can work towards reducing school absenteeism and improving overall oral health outcomes.

## Limitations

Limitations of the study included potential biases associated with self-reported data, the young age of the participants, which may have impacted the accuracy of responses, and the limited duration of the study, which may have restricted the depth of data collection and analysis.

## Implications

The implications of this study are significant for addressing oral health issues among school-going children in AlKharj city, Saudi Arabia. The formulation and implementation of awareness programs based on the findings of this study represent a proactive approach to improving oral health outcomes and overall well-being. By educating children about the importance of preventive measures against oral diseases, such as regular brushing, flossing, and dental check-ups, we can empower them with the knowledge and skills needed to maintain good oral hygiene. Moreover, the awareness programs also highlighted the importance of healthy eating habits and lifestyle choices in supporting overall academic success. By emphasizing the link between proper nutrition, lifestyle, and academic performance, we can encourage children to make healthier choices and adopt positive habits that contribute to their long-term well-being. These awareness programs have the potential to instill lasting behavioral changes among children, leading to improved oral health outcomes and overall quality of life. Additionally, by targeting children at a young age, we can lay the foundation for lifelong oral health habits and reduce the burden of oral diseases in the future.

## Conclusion

The study highlighted a critical gap in oral health awareness among school children, underscoring the need for targeted interventions. While the oral hygiene educational program has been implemented and future data will shed light on its effectiveness, preliminary observations suggest that such programs could potentially improve oral health outcomes and overall engagement among students.

## Competing interests

## Data Availability

The datasets used and analysed during the current study are available from the corresponding author on reasonable request.

## References

[1] Peng X, et al. Oral microbiota in human systematic diseases. Int J Oral Sci. 2022;14(1):14. 35236828 10.1038/s41368-022-00163-7 PMC8891310

[2] Abebe GM. Oral biofilm and its impact on oral health, psychological and social interaction. Int J Oral Dental Health. 2021;7:127.

[3] McQuilken SA. The mouth, stomach and intestines. Anaesth Intens Care Med. 2021;22(5):330–5. 10.1016/j.mpaic.2021.04.001

[4] Ptasiewicz M, et al. Armed to the teeth—the oral mucosa immunity system and microbiota. Int J Mol Sci. 2022;23(2):882. 35055069 10.3390/ijms23020882 PMC8776045

[5] Şenel S. An overview of physical, microbiological and immune barriers of oral mucosa. Int J Mol Sci. 2021;22(15):7821. 34360589 10.3390/ijms22157821 PMC8346143

[6] Moin, M., et al. *The association of socioeconomic and lifestyle factors with the oral health status in school-age children from Pakistan: a cross-sectional study*. In *Healthcare*. 2023. MDPI. 10.3390/healthcare11050756 PMC10001539 36900761

[7] Achmad, H., et al., *Literature review: Problems of dental and oral health primary school children.* Indian Journal of Forensic Medicine & Toxicology, 2021. **15**(2).

[8] Rao, K.Y., *Oral health status of school children aged 13–15 years in tumkur district*. 2009, Rajiv Gandhi University of Health Sciences (India).

[9] Imane, B., et al., *Prevalence and Antibiotic Susceptibility Pattern of Oral Pathogens Causing Dental Caries in Medea (Algeria).* South Asian Journal of Experimental Biology, 2023. **13**(2).

[10] Amato JN, et al. Examining the relationship between social and school environment and children’s caries experience using primary and secondary data: A cluster analysis. Caries Res. 2021;55(2):79–87. 33601379 10.1159/000513256

[11] Kumar, V.S., et al., *Association of Dental Caries and Oral Health Impact Profile in 12-Year-Old School Children: A Cross-Sectional Study.* Journal of Clinical & Diagnostic Research, 2018. **12**(9).

[12] Organization, W.H., *World Oral Health Report. Published 2018*. 2020.

[13] Bardi M, et al. Activities for engaging schools in health promotion. Health Educ. 2014;114(4):271–80. 10.1108/HE-08-2013-0040

[14] Batwala, B., et al., *Oral health status of school children in Mbarara, Uganda.* African health sciences, 2007. **7**(4). PMC3074375 21499489

[15] Shung-King M, et al. School health in South Africa: reflections on the past and prospects for the future. South African Health Reviewkkk. 2013;2013(1):59–71.

[16] Halvari AEM, et al. Oral health and dental well-being: Testing a self-determination theory model. J Appl Soc Psychol. 2013;43(2):275–92. 10.1111/j.1559-1816.2012.00996.x

[17] Bleakley, H., *Child Health and Educational Outcomes.* Education Policy in Developing Countries, 2013: p. 107.

[18] Gupta, V., et al., *Assessment of Oral Hygiene Practices and Awareness of Periodontal-Systemic Health Interrelationship Amongst the Local Population of Kanpur Region-A Cross Sectional Study.* Journal of Oral Health & Community Dentistry, 2016. **10**(1).

[19] Kuppuswamy VL, et al. Oral hygiene status, knowledge, perceptions and practices among school settings in rural South India. Oral Health Dental Manage. 2014;13(1):146–54. 24603932

[20] Usha G, et al. Validity and reliability of oral impacts on daily performances frequency scale: a cross-sectional survey among adolescents. J Clin Pediatr Dent. 2012;36(3):251–6. 22838226 10.17796/jcpd.36.3.x563pl1q66u7l068

[21] Moses J, et al. Prevalence of dental caries, socio-economic status and treatment needs among 5 to 15 year old school going children of Chidambaram. J Clin Diagn Res. 2011;5(1):146–51.

[22] Al Suwyed AS, et al. The silent epidemic of common oral diseases among the Arab population: An emerging health problem. J Family Med Primary Care. 2021;10(8):2768–74. 34660403 10.4103/jfmpc.jfmpc_323_21 PMC8483081

[23] Siddiqui, A.A., et al., *Oral health in Saudi Arabia.* Handbook of Healthcare in the Arab World, 2021: p. 3511–3536.

[24] Sabbagh HJ, et al. Oral health needs and barriers among children in Saudi Arabia. Int J Environ Res Public Health. 2022;19(20):13584. 36294162 10.3390/ijerph192013584 PMC9603417

[25] Silveira MR. *Epidemiology of dental caries in the world.* Oral Health Care—Pediatric. Res Epidemiol Clin Pract. 2012;8:149–68.

[26] Al-Jobair AM, et al. Medical and dental health status of orphan children in central Saudi Arabia. Saudi Med J. 2013;34(5):531–6. 23677271

[27] Agaku IT, et al. Association between unmet dental needs and school absenteeism because of illness or injury among US school children and adolescents aged 6–17 years, 2011–2012. Prev Med. 2015;72:83–8. 25575801 10.1016/j.ypmed.2014.12.037

[28] Jackson SL, et al. Impact of poor oral health on children’s school attendance and performance. Am J Public Health. 2011;101(10):1900–6. 21330579 10.2105/AJPH.2010.200915 PMC3222359

[29] Collaborators GOD, et al. Global, regional, and national levels and trends in burden of oral conditions from 1990 to 2017: a systematic analysis for the global burden of disease 2017 study. J Dent Res. 2020;99(4):362–73. 32122215 10.1177/0022034520908533 PMC7088322

[30] Organization, W.H., *Global oral health status report: towards universal health coverage for oral health by 2030. Regional summary of the African Region*. 2023: World Health Organization.

[31] Purohit BM, et al. Universal oral health coverage–perspectives from a developing country. Int J Health Plann Manage. 2022;37(2):610–8. 34704290 10.1002/hpm.3361

[32] Samuel SR, et al. School interventions–based prevention of early-childhood caries among 3–5-year-old children from very low socioeconomic status: Two-year randomized trial. J Public Health Dent. 2020;80(1):51–60. 31710096 10.1111/jphd.12348

[33] BINDU, A.S., *Influence of Socioeconomic Status on Oral Health Among School Going Children Aged 12–15 Years in Davangere City-A Cross-Sectional Study*. 2018, Rajiv Gandhi University of Health Sciences.

[34] Alyafei, N.A.R.J., *Asnani’–my teeth–exploring behavioural prevention strategies for dental caries in Qatari primary schools: A case study approach*. 2021: Bangor University (United Kingdom).

[35] Samim, F., *Dental health needs and related factors in inner-city Vancouver elementary school-aged children*. 2013, University of British Columbia.

[36] Petersen PE. Global policy for improvement of oral health in the 21st century–implications to oral health research of World Health Assembly 2007, World Health Organization. Commun Dent Oral Epidemiol. 2009;37(1):1–8. 10.1111/j.1600-0528.2008.00448.x 19046331

[37] El Tantawi M, et al. Prevalence and data availability of early childhood caries in 193 United Nations Countries, 2007–2017. Am J Public Health. 2018;108(8):1066–72. 29927650 10.2105/AJPH.2018.304466 PMC6050821

[38] Alsumait A, et al. Impact of dental health on children’s oral health-related quality of life: a cross-sectional study. Health Qual Life Outcomes. 2015;13:1–10. 26149439 10.1186/s12955-015-0283-8 PMC4491877

[39] Mohd Khairuddin AN, et al. Impact of dental visiting patterns on oral health: A systematic review of longitudinal studies. BDJ open. 2024;10(1):18. 38448428 10.1038/s41405-024-00195-7 PMC10917741

[40] Shivakumar K, et al. Oral health status and dental treatment needs of sensory-impaired children of Satara District. India J Int Oral Health. 2017;9(5):197–201. 10.4103/jioh.jioh_158_17

[41] Togoo RA, et al. Prevalence of first permanent molar caries among 7–10 years old school going boys in Abha city, Saudi Arabia. J Int Oral Health. 2011;3(5):29.

[42] Mustafa M, et al. Extent of awareness regarding oral health and dental treatment needs among individuals with hearing and speech impairments in Saudi Arabia. J Int Soc Prevent Commun Dent. 2018;8(1):70–6. 10.4103/jispcd.JISPCD_194_17 PMC5853046 29629332

[43] Farsi N, et al. Caries risk assessment in preschool children in Saudi Arabia. Oral Health Prev Dent. 2013;11(3):271–80. 23957045 10.3290/j.ohpd.a30479

